# Reduction in HER-2 protein expression in a breast tumor HER-2 positive after only one injection of Trastuzumab: a case report

**DOI:** 10.4076/1757-1626-2-8099

**Published:** 2009-06-09

**Authors:** Ibrahim Elghissassi, Hanane Inrhaoun, Stephane Vignot, Hind Mrabti, Hassan Errihani

**Affiliations:** 1 Department of Medical Oncology, National institute of OncologyRabatMorocco; 2 Department of Medical Oncology, Pitié-Salpêtrière Hospital GroupParisFrance

## Abstract

HER-2 is overexpressed in 20 to 30% of breast cancer. Generally, metastases of a breast tumor have the same HER-2 status, although some discordances were reported.

We report a case of reduction in HER-2 expression assessed by immunohistochemistry, following one day of a Trastuzumab injection, of a metastatic breast cancer to lymph nodes. Initially, the breast tumor HER-2 status was positive according to the same technique.

We raise a hypothesis about technical interference and discuss the case in the framework of the existing literature.

## Introduction

HER-2 is a member of the ErbB family of transmembrane growth factor receptors with tyrosine kinase activity. Amplification of the *HER-2* gene or overexpression of the HER-2 protein is associated with adverse disease prognosis and a worse response to treatment and has been identified in 20-30% of primary breast carcinomas [1-3]. Its determination can be done by an immunohistochemical (IHC) assay or fluorescence *in situ* hybridisation (FISH) [4,5].

Trastuzumab is a humanized monoclonal anti-HER-2 antibody. Its efficacy is associated directly with the HER-2 status of the breast tumor. Indeed, the benefit of trastuzumab is clearly limited to those patients with HER-2 overexpression or amplification [6].

Generally, metastases of a breast tumor have the same HER-2 status [7], although some discordances were reported [8]. We report a case of reduction in HER-2 expression assessed by IHC, following one day of a Trastuzumab injection, of a tumor which was positive before according to the same technique raising a hypothesis about technical interference

## Case presentation

It is about a 66 year old Caucasian woman who presented in 1982 a breast adenocarcinoma T2N0M0 treated by lumpectomy and radiotherapy. In 1989, she presented a rectal adenocarcinoma T4N0M0 treated by surgery.

Fifteen years later, the appearance of a left supra-clavicular lymph node justified a biopsy showing metastases of breast adenocarcinoma hormonal receptor negative and HER-2 protein positive (expression was +++) assessed by IHC. She had also infra and sub-diaphragmatic lymph node involvement. A progression was noted after 4 cycles of chemotherapy consisting of Docetaxel 75 mg/m^2^ intravenously on day 1 and Doxorubicin 60 mg/m^2^ intravenously on day 1 repeated every three weeks.

In order to exclude an associated rectal recurrence, a biopsy of a retroperitoneal lymph node was programmed. Monotherapy by Trastuzumab 4 mg/kg intravenously was undertaken the day before biopsy. Anatomopathological analysis with IHC confirms breast origin and shows identical aspect with the left supra-clavicular lymph node but the HER-2 status was negative (expression was only +). Shortly after, the patient died due to progressive disease.

## Discussion

In this case, the reduction in HER-2 protein expression can not be explained by the elimination of expressing HER-2 clones, the patient having received only one injection of Trastuzumab. Also, it can not be explained by chemotherapy that received patient. It is currently known that chemotherapy would not modify the HER-2 status in metastatic lesions [7].

Tumor heterogeneity can be evoked. The main targets of any therapy in metastatic breast cancer are the metastases. However, in the great majority of cases, HER-2 status is determined on the primary tumour, and there are few published data regarding the comparison of HER-2 status between the primary and the metastatic sites and between multiple distant metastatic sites from the same patient and all have reported a high level of consistency although not complete [7-9]. The discordances can be explained by the fact that in several of these studies, HER-2 status analysis was made by IHC only. Another possible explanation may be sample's collection which could be done many years before the present analysis, and therefore some protein degradation might have occurred.

Tumor HER-2 status is generally assessed as protein overexpression by IHC while *HER-2* gene amplification is detected by FISH and recently by CISH (Chromogenic in situ hybridization) [10]. It is known that there is a high level of correlation between FISH and IHC in the evaluation of HER-2 status of breast cancers and thus, it is not recommended to make a FISH analysis when HER-2 status is negative by IHC [11].

In our case, we raise a hypothesis about technical interference related to the injection of Trastuzumab the day before biopsy involving a reduction in HER-2 IHC expression and thus we recommend, in such cases, to make a study by FISH on the same biopsy which can determine HER-2 status pertinently. This deserves to be elucidated by studies with large series.

## List of abbreviations

CISH: Chromogenic in situ hybridization
FISH: fluorescence *in situ* hybridization
HER2: Human epidermal growth factor receptor 2
IHC: immunohistochemical
